# New Typical Vector of Neurotoxin β-*N*-Methylamino-l-Alanine (BMAA) in the Marine Benthic Ecosystem

**DOI:** 10.3390/md14110202

**Published:** 2016-11-04

**Authors:** Aifeng Li, Jialiang Song, Yang Hu, Longji Deng, Ling Ding, Meihui Li

**Affiliations:** 1 College of Environmental Science and Engineering, Ocean University of China, Qingdao 266100, China; sjl0320@163.com (J.S.); ouc_huyang@163.com (Y.H.); denglongjicool@126.com (L.D.); wddingling@163.com (L.D.); limeihui0211@163.com (M.L.); 2 Key Laboratory of Marine Environment and Ecology, Ocean University of China, Ministry of Education, Qingdao 266100, China

**Keywords:** β-*N*-methylamino-l-alanine (BMAA), 2,4-diaminobutyric acid (DAB), *Neverita didyma*, HILIC-MS/MS, marine ecosystem

## Abstract

The neurotoxin β-*N*-methylamino-l-alanine (BMAA) has been identified as an environmental factor triggering neurodegenerative diseases such as Amyotrophic Lateral Sclerosis (ALS) and Alzheimer’s disease (AD). We investigated the possible vectors of BMAA and its isomers 2,4-diaminobutyric acid (DAB) and *N*-2(aminoethyl)glycine (AEG) in marine mollusks collected from the Chinese coast. Sixty-eight samples of marine mollusks were collected along the Chinese coast in 2016, and were analyzed by an HILIC-MS/MS (hydrophilic interaction liquid chromatography with tandem quadrupole mass spectrometer) method without derivatization. BMAA was detected in a total of five samples from three species: *Neverita didyma*, *Solen strictus*, and *Mytilus coruscus*. The top three concentrations of free-form BMAA (0.99~3.97 μg·g^−1^ wet weight) were detected in *N. didyma*. DAB was universally detected in most of the mollusk samples (53/68) with no species-specific or regional differences (0.051~2.65 μg·g^−1^ wet weight). No AEG was detected in any mollusk samples tested here. The results indicate that the gastropod *N. didyma* might be an important vector of the neurotoxin BMAA in the Chinese marine ecosystem. The neurotoxin DAB was universally present in marine bivalve and gastropod mollusks. Since *N. didyma* is consumed by humans, we suggest that the origin and risk of BMAA and DAB toxins in the marine ecosystem should be further investigated in the future.

## 1. Introduction

BMAA (β-*N*-methylamino-l-alanine), as an unusual amino acid, has been hypothesized to trigger the pathogenesis of neurodegenerative diseases like Amyotrophic Lateral Sclerosis (ALS) and Alzheimer’s disease (AD) [1,2]. Extensive in vitro experiments have demonstrated that the neurotoxicity of BMAA for neurons is a result of multiple mechanisms including action at NMDA (*N*-methyl-d-aspartic acid) or AMPA (α-amino-3-hydroxy-5-methyl-4-isoxazole propionic acid)/kainate receptors, generation of ROS (reactive oxygen species), and production of excessive dopamine [3,4,5,6,7]. Recent reports have documented that BMAA inhibited antioxidant enzymes of the macrophyte *Ceratophyllum demersum* and of aquatic plants (*Lomariopsis lineata*, *Fontinalis antipyretica*, *Riccia fluitans*, and *Taxiphyllum barbieri*), indirectly implicating the induction of oxidative stress in plants by BMAA [8,9]. The accumulation of BMAA in zooplankton results in a loss of swimming ability, phototaxis, and reproductive ability, which may also be linked to toxicity due to oxidative stress [10,11,12]. Neuro-muscular and developmental abnormalities of zebrafish (*Danio rerio*) embryos occurred because of the exposure to BMAA [13].

A typical bio-magnification pathway of BMAA has been reported in the food chains within the island of Guam [1]. BMAA originates within diverse taxa of cyanobacteria living in terrestrial, as well as freshwater, brackish, and marine environments [14,15]. Since cyanobacteria represent a large proportion of the base of the food chain in marine ecosystems, further research is needed to understand their importance and bio-magnification pathways.

**Figure 1:**
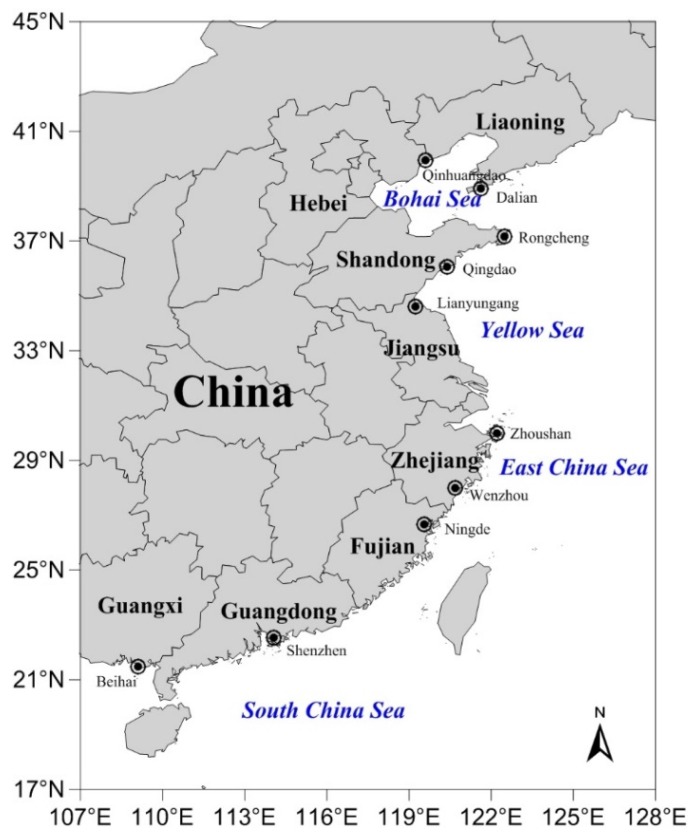
Sampling locations for marine mollusk animals collected in March 2016 and analyzed for β-*N*-methylamino-l-alanine (BMAA), 2,4-diaminobutyric acid (DAB) and *N*-2(aminoethyl)glycine (AEG) in this study (samples were collected from aquaculture zones in Zhoushan and Shenzhen cities, the other samples were purchased from local seafood markets in other eight cities).

In addition to cyanobacteria as an originating source of BMAA, a marine dinoflagellate (*Heterocapsa triquetra*) and diatoms (*Achnanthes* sp., *Navicula pelliculosa*, *Skeletonema marinoi*, *Thalassiosira* sp., *Proboscia inermis*) have also been implicated [16,17]. A picture is developing of widespread occurrence of BMAA in aquatic organisms (Table S1) [18,19,20,21,22,23,24,25,26,27,28,29,30,31,32,33,34,35,36,37,38]. Of particular interest is the presence of BMAA in marine animals collected from the coastal area of Florida, USA, and the southern coast of Stockholm, Sweden [22,23]. Furthermore, BMAA was also detected in samples of mussels and oysters cultivated in seawater using LC-MS/MS analysis of underivatized extracts [35,36,37]. However, the controversy regarding data on BMAA in aquatic ecosystems has also been reviewed by Faassen [39], who demonstrated that it is still need to develop and validate the precise analytical methods for BMAA in the future. Based on these new findings, contamination with BMAA and its isomers 2,4-diaminobutyric acid (DAB) and *N*-2(aminoethyl)glycine (AEG) was investigated in diverse mollusks collected from ten cities along the Chinese coast in this study (Figure 1). It is the objective of the present study to assess the risk of the neurotoxin BMAA in marine mollusks in order to protect seafood safety and consumer health.

## 2. Results

**Figure 2:**
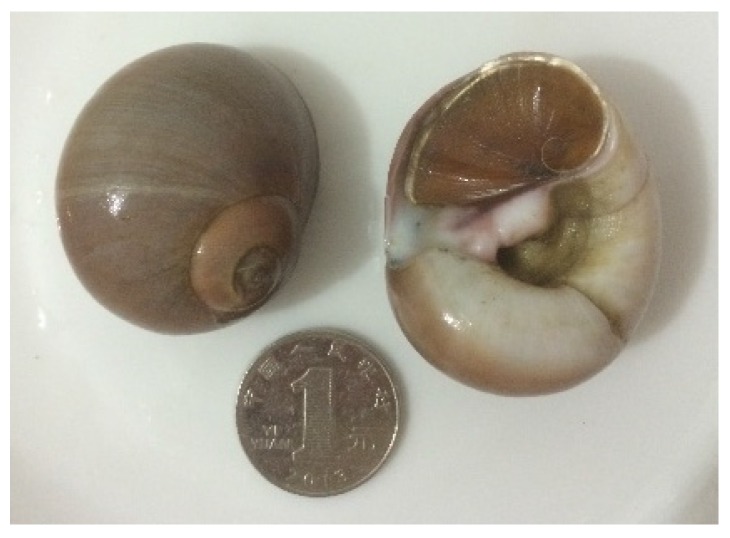
Photograph of wild benthic gastropod *Neverita didyma* as a typical vector of neurotoxin BMAA in Chinese marine ecosystem.

**Figure 3:**
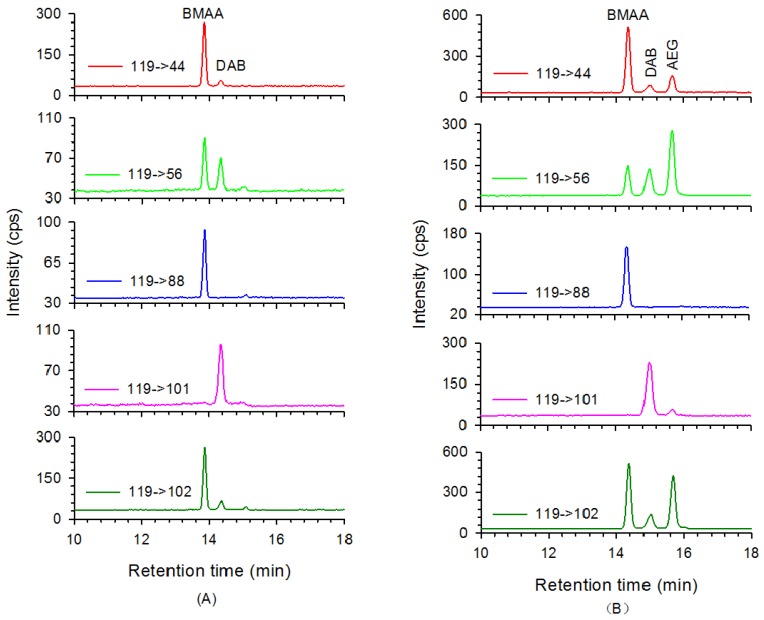
HILIC-MS/MS chromatograms for extract of gastropod *Neverita didyma* (**A**); and its extract spiked with BMAA, DAB and AEG standards (**B**).

BMAA was detected in a total of five samples from three marine species collected from Chinese coastal waters. DAB was detected in 78% of the samples (53/68) representing 23 species in this research (Table 1). The highest concentrations of BMAA were detected in the same species of benthic gastropod *Neverita didyma* collected from Dalian, Rongcheng, and Lianyungang cities in March 2016, respectively (Figure 2). BMAA was also detected in two additional samples of *N. didyma* collected from Laizhou and Qingdao cities. Neither BMAA nor DAB was detected in the protein of any mollusk species. The identity of BMAA was confirmed in the samples of *N. didyma* using an extract spiked with mixed standards which was then compared with the original extract of *N. didyma* (Figure 3). The intensity of the selective reaction monitoring (SRM) transitions (measured as a percent of the total peak areas of four qualitative transitions) was used to identify BMAA, DAB, and AEG as compared to standard solutions, the original extract, and spiked extracts of *N. didyma* (Table 2). HILIC-MS/MS chromatograms for original extract and spiked extract of *N. didyma* clearly demonstrated that the BMAA and DAB were present (Figure 3). Retention times of BMAA, DAB, and AEG peaks slightly shifted in different extracts of mollusk samples due to the effect of matrix on the HILIC column separation. Concentrations of 16 protein amino acids in three samples of gastropod *N. didyma* were also determined (Table S2).

## 3. Discussion

A highly selective and sensitive HILIC-MS/MS method for BMAA, DAB, and AEG was used to analyze mollusk samples collected from the Chinese coast in this study. Underivatized extracts were analyzed and four SRM transitions were used to distinguish each isomer. The neurotoxin BMAA was detected in five mollusk samples collected in the first batch investigated and two additional gastropod samples of *N. didyma*. DAB, on the other hand, was found in 53 of 68 samples (0.051~2.65 μg·g^−1^ wet weight), and AEG was not observed in any sample (Table 1). Two lower concentrations of BMAA were quantified in samples of *Mytilus coruscus* (Zhoushan City) and *Solen strictus* (Rongcheng City) at 0.45 and 0.66 μg·g^−1^ wet weight, respectively. BMAA concentrations ranged from 0.86 to 3.97 μg·g^−1^ wet weight of *N. didyma* in five samples collected from different cities. The highest detection rate of BMAA (100%) suggests that *N. didyma* is a typical vector of BMAA in the marine benthic ecosystem. A comparison of the relative intensity of different SRM transitions for these compounds in standard solution, original and spiked extracts showed that the peaks of BMAA and DAB detected in *N. didyma* were identical to both standard compounds (Table 2). Comparison of HILIC-MS/MS chromatograms demonstrated that the standard BMAA and DAB completely overlap with the peaks detected in the original extract of *N. didyma* (Figure 3). Retention times of BMAA, DAB, and AEG peaks slightly shifted in different extracts of mollusk samples due to the effect of matrix on the HILIC column separation. Retention times of BMAA, DAB, and AEG obviously shortened (~1.0 min) in the matrix of gastropod (Table 2). Intensity ratio of different transitions should be considered as another critical requirement for qualitative analysis of these isomer compounds. Both mollusks, *M. coruscus* and *S. strictus*, filter-feed on phytoplankton cells and other organic particles, but the former species is cultured using lifting rope and the latter species is grown in benthic environments. In contrast, the gastropod *N. didyma* is a predatory sea snail that lives in the benthic environment and eats other bivalves such as razor shell and clams. Thus, the *N. didyma* is regarded as a natural enemy for artificially cultured benthic bivalves. It is also a popular seafood for residents living close to the northern coast of China due to valuable nutrient composition. Fish of *Navodon* spp. are known to eat *N. didyma* in natural environments, however, no analysis of BMAA bio-magnification has been included for this species. All seafood products of *N. didyma* sold in markets are captured from wild benthic environments and are, therefore, a possible BMAA exposure route for humans. The transfer of BMAA between trophic levels was implied by its simultaneous detection in razor shell *S. strictus*, food source, and predator, *N. didyma*, collected from Rongcheng City. However, in order to confirm trophic transfer one would want to have more BMAA positive samples of *S. strictus* as well as experimental data. 

Other research groups have universally detected BMAA in mussel samples (*Mytilus galloprovincialis*) and oysters (*Crassostrea gigas*) collected from a French marine lagoon in concentrations ranging from 0.20 to 6.7 μg·g^−1^ dry weight of digestive gland tissues [24,38]. Only small variations of BMAA and DAB levels were observed and these were not correlated to any of the phytoplankton species reported but the concentrations were consistent between two methods, one using AQC (6-aminoquinolyl-*N*-hydroxysuccinimidyl carbamate) derivatization and the other using HILIC [24,38]. BMAA was also detected in two mussel tissues (0.95 and 1.20 μg·g^−1^) used for reference materials for marine algal toxins in National Research Council-Canada using a sensitive and highly selective multidimensional HILIC-DMS-MS/MS method [36]. However, no BMAA was found in 7 samples of *Mytilus galloprovincialis* or in 10 samples of *Crassostrea* sp. collected from the Chinese coast in this study. Only a small concentration of BMAA was detected in one sample of mussel *M. coruscus* cultured in Gouqidao aquaculture zone in Zhoushan City, but it did not appear in *M. coruscus* collected from Wenzhou City. The typical vector of BMAA in Chinese coast may be different than that in French waters. We did not detect BMAA accumulation within diverse filter-feeding bivalves in the Chinese marine ecosystem. The BMAA concentrations were converted to 4.30~19.85 μg·g^−1^ dry weight of whole soft tissues of *N. didyma*, assuming the tissue water percent is about 80%. The free form of BMAA detected in the muscle and gland tissues of *N. didyma* collected from Laizhou City was 0.36 and 0.95 μg·g^−1^ wet weight, respectively. The BMAA concentration in gland tissue was significantly higher than that of muscle tissue in the gastropod *N. didyma*. 

**Figure 4:**
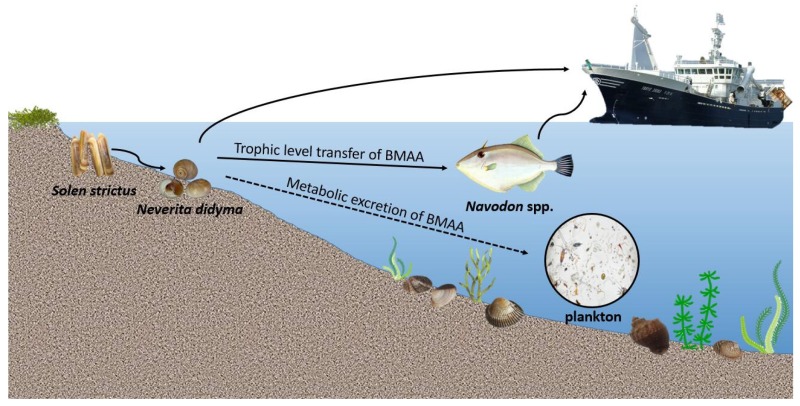
Possible pathways for the exposure of human and marine organisms to the BMAA accumulated by gastropod *Neverita didyma*.

BMAA has been testified as an important environmental factor triggering neurodegenerative diseases such as ALS and AD [40]. In previous studies, BMAA has been shown to be misincorporated into proteins by human neuroblastoma cells and fibroblasts, resulting in the formation of auto-fluorescent material and causing the induction of apoptotic cell death [41]. It has been shown that l-serine may be able to prevent this development of misfolded proteins [42] and reduce the density of neurofibrillary tangles caused by BMAA [40]. Amino acids were also analyzed in muscle and gland tissues of three samples of *N. didyma* collected from Dalian, Rongcheng, and Laizhou cities. Results showed that the concentration of 16 amino acids were similar in muscle tissues of three samples (all values with relative standard deviation (RSD) below 10%), but were more variable in gland tissues (RSD values of 6 amino acids were above 10%) (Table S2). The components of protein amino acids in gland tissues of *N. didyma* should be easily affected by food composition, but should also be relatively stable in muscle tissues. Therefore, the amino acids in muscle tissue might provide information relevant to the discussion of the effect of BMAA on protein synthesis within *N. didyma*. We have checked the recoveries of BMAA spiked in the residual protein pellets after extraction of free-form toxin and compared them before and after SPE purification. The recovery of BMAA spiked in the protein pellets of gastropod *Rapana venosa* at concentrations of 0.2 and 1.0 μg·g^−1^, was 15.0% and 17.7%, respectively, before SPE purification. The recovery of BMAA spiked in the scallop *Chlamys farreri* was 29.3% and 32.2%, respectively. However, the recovery of BMAA spiked in the protein pellets of gastropod and scallop at a concentration of 1.0 μg·g^−1^, reduced by 5–6 times after SPE purification, which demonstrated that an obvious loss of BMAA occurred in the SPE purification process. There is still a need to explore the reasons in the future although no protein-bound BMAA was found in the hydrolyzed extracts of gastropod before or after SPE purification. Since no protein-bound BMAA was detected in these samples, it is possible that the neurotoxin BMAA did not impact protein synthesis in *N. didyma*. However, concentrations of nine amino acids (Arg, Asp, Glu, Leu, Lys, Ser, Thr, Ile, and Met) in muscle tissues reduced with the rise of BMAA concentration in the gastropod *N. didyma*. This phenomenon suggests that BMAA affected the synthesis of protein through some pathways although it did not misincorporate into proteins. Alternatively, protein misincorporation was infrequent and below our ability for detection. BMAA toxicity in aquatic plants and animals has been previously demonstrated [8,9,10,11,13]. Oxidative stress may be a universal pathway across different species of aquatic organisms. Perhaps the free form of BMAA, like other soluble algal toxins, could be excreted into seawater by metabolism of vectors. Possible pathways for the exposure of human and marine organisms to BMAA in marine ecosystem have been speculated (Figure 4). The finding of BMAA in marine ecosystem suggests that further research should be conducted in this area in order to protect human health. 

## 4. Experimental Section

### 4.1. Chemicals

BMAA (L-BMAA hydrochloride, B107, 10 mg) and dl-2,4-diaminobutyric acid dihydrochloride (DAB, D3758, 1 g) standards were purchased from Sigma-Aldrich (Steinheim, Germany). Standard *N*-2(aminoethyl)glycine (AEG, A608975, 1 g) was purchased from Toronto Research Chemicals Inc. (Toronto, ON, Canada). A mixed standard of BMAA (1.0 mg·mL^−1^), DAB (1.0 mg·mL^−1^), and AEG (1.0 mg·mL^−1^) was prepared in 2 mmol·L^−1^ hydrochloric acid (HCl). The standard was diluted in 2 mmol·L^−1^ HCl to prepare a series of working solutions for LC-MS/MS calibration (0.005, 0.05, 0.25, 0.5, 1.25, and 5.0 μg·mL^−1^). Methanol and acetonitrile purchased from Merck were high-performance liquid chromatography (HPLC) grade. HCl and ammonium hydroxide were purchased from Sinopharm Chemical Reagent. Formic acid (FA) and trichloroacetic acid (TCA) were obtained from Sigma-Aldrich (Steinheim, Germany). Water used in these experiments was purified by a Milli-Q ultrapure water system (Millipore, Shanghai, China) to a quality of 18 MΩ·cm or better.

### 4.2. Samples and Sampling Locations

Many large aquaculture zones for growing shellfish, such as mussels, scallops, and oysters are located along Chinese coasts. Ten sampling locations were selected to collect diverse marine mollusks in March 2016 (Figure 1). With the exception of seven mollusk samples which were directly obtained from the aquaculture zones in Zhoushan and Shenzhen cities, the samples were purchased from local seafood markets. A total of 68 samples attached to 29 species and 28 genera of marine mollusks were collected here. More samples of some popular seafood products including *Crassostrea* sp. (10), *Mytilus galloprovincialis* (7), and *Ruditapes philippinarum* (5) were collected because they are important economic mollusk species cultured in China. An additional two samples of wild gastropod *Neverita didyma* (Figure 2) were collected from Laizhou and Qingdao cities on 26 April and 22 May, 2016, respectively, after the batch investigation.

### 4.3. Sample Preparation

All mollusk samples were stored with shells at −20 °C before extraction. After removing shellfish shells the whole soft tissues of 3~10 animal individuals were treated with a tissue mincer due to their body sizes. Mollusk tissues were extracted by mixing 1.0 g of homogenate with 3 mL of 0.1 M TCA for 4 min using a blender in a 10 mL centrifuge tube to extract free-form of BMAA, DAB and AEG. After centrifuging (8000× *g*, 10 min) the supernatant was decanted to a 10 mL flask and the tissue was extracted again for 3 min using 3 mL of 0.1 M TCA. This supernatant was combined with that of the first step, and the extraction step was repeated one final time. The combined supernatants were made up to 10 mL using 0.1 M TCA and an aliquot solution was filtered (0.22 μm) before analysis.

After the extraction for free form of BMAA, the residual pellet was transferred to a glass vial and mixed with 3 mL of 6 M HCl used to wash the centrifuge tube. Mixture was vortexed for 4 min and then heated at 110 °C for 24 h. Following hydrolysis, 3 mL of pure water was added and the solution was centrifuged at 8000× *g* for 10 min. The supernatant was transferred into a glass vial and mixed with 5 mL of pure water. Then, the mixture solution was transferred into a glass beaker using funnel and filter paper because there were still some fine particles after centrifugation; 6 mL of pure water was used to wash the glass vial and then combined with the supernatant. An additional 9 mL and 4 mL of pure water was used to wash filter paper and funnel, respectively. Then 30 mL of pure water was added to this mixture in order to dilute the 6 M HCl solution about 20 times before purification. 

The hydrolyzed solution was purified by Oasis MCX (3 cc, 60 mg) SPE cartridges according to the procedures published in previous study [34]. SPE cartridges were activated using 2 mL of methanol, and 2 mL of pure water was used to equilibrate the cartridges. The extract solution of BMAA was loaded using a slow rate below 1 mL·min^−1^. Then 2 mL of 0.1 M HCl and 2 mL of methanol was used to orderly wash the cartridge. Finally, 2 mL of 5% NH_4_OH was used to elute BMAA and collected in glass vials. The eluent was blow-dried under nitrogen at 50 °C and then reconstituted in 1.0 mL of 20 mmol·L^−1^ HCl. The solution was filtered through 0.22 μm nylon membrane before analysis.

Additionally, standard BMAA was also spiked in the protein pellets of gastropod *R. venosa* and scallop *C. farreri* at concentrations of 0.2 and 1.0 μg·g^−1^, respectively, in order to determine the recoveries of BMAA before and after SPE purification. One gram of free-form BMAA homogenate was extracted and the residual pellet was spiked with 200 μL of 1.0 and 5.0 μg·mL^−1^ standard BMAA, respectively. Then 2 mL of 6 M HCl was used for hydrolysis at 110 °C for 24 h. After centrifuging the supernatant was transferred into a glass beaker using funnel and filter paper washed with about 8 mL of pure water. The filter solution was lyophilized and then was reconstituted in 10 mL of 20 mmol·L^−1^ HCl. The recoveries of BMAA in this extract were analyzed before and after SPE purification addressed above.

### 4.4. LC-MS/MS Analysis

All samples were analyzed by an Agilent 1290 HPLC (Palo Alta, CA, USA) coupled with an Agilent 6430 mass spectrometer with an electro-spray ionization source. A 5-μm TSK Gel Amide-80^®^ hydrophilic interaction liquid chromatography (HILIC) column (250 mm × 2 mm internal diameter, Tosoh Bioscience LLC) was maintained at 40 °C to separate BMAA, DAB, and AEG, using a binary mobile phase system composed of water (solvent A) and 95% acetonitrile (solvent B) each with 50 mmol·L^−1^ FA. The gradient was linear from 90% to 60% solvent B over 15 min and was subsequently held for 4 min, decreased to 55% B at 19.01 min, and held again for 7.99 min before re-equilibration. The flow rate was set at 350 μL·min^−1^, and an injection volume of 5 μL was used throughout. The MS was operated in positive ion mode with detection by the selective reaction monitoring (SRM) mode to quantitate BMAA, DAB, and AEG. Five transitions including 119 → 102, 119 → 101, 119 → 88, 119 → 56, and 119 → 44 were monitored at collision energies (CE) of 8, 5, 8, 15, and 20 V, respectively. The electro-spray voltage was set to 5500 V with a source temperature of 450 °C. Nitrogen was used for the nebulizer and curtain gases. The *m*/*z* 119 → 44, 119 → 101, and 119 → 102 transitions were used to quantitate BMAA, DAB, and AEG, respectively. Simultaneously, four transitions were used as qualitative signals for each toxin. The signal intensity ratio between different SRM transitions was used as a criterion to confirm these toxin analogues. The limits of detection (LOD) for BMAA and AEG (154 and 52 pg, respectively), were determined using standard solution of 0.05 μg·mL^−1^ when the signal to noise ratio (S/N) in four transitions used for qualitative diagnosis was above 3:1. The LOD for DAB (S/N = 3, 6.7 pg) was determined in standard solution of 0.005 μg·mL^−1^. According to the extraction ratio 1:10 (1 g/10 mL), the LOD for BMAA, DAB, and AEG in mollusk samples were calculated as 0.31, 0.013, and 0.10 μg·g^−1^ wet weight, respectively. The qualitative criteria for these toxins were very critical due to four different transitions synchronously used for every toxin. 

### 4.5. Amino Acid Analysis

Five specimens of *N. didyma* collected from Dalian, Rongcheng, and Laizhou cities were respectively anatomized into muscle and gland tissues to analyze amino acids. Weight of 0.3 g homogenate tissue was mixed with 10 mL of 6 M HCl and hydrolyzed for 24 h at 110 °C. After the hydrolyzed solution was cooled to room temperature, it was filtered into a 50 mL volumetric flask and was made to the scale using pure water. Part of 2 mL diluted solution was blow-dried under nitrogen and then reconstituted in 2 mL of 20 mmol·L^−1^ HCl. Then an automatic amino acid analyzer L-8800 (Hitachi, Tokyo, Japan) was used to determine amino acids. 

## 5. Conclusions

The benthic gastropod *Neverita didyma* has been identified as a new vector of BMAA in the Chinese marine ecosystem. This is the first report of a mollusk gastropod identified as a new animal class contaminated by the neurotoxin BMAA. BMAA exposure mediated by the gastropod *N. didyma* could affect the health of marine organisms and human consumers which warrants further investigation. BMAA was not detected within protein tissues of gastropods or bivalves, although it is possible that BMAA influences protein synthesis within mollusks. In line with other research, DAB was universally detected in most of the diverse marine mollusks without species-specific or regional differences. DAB may also be a potential environmental risk factor affecting human health and seafood safety.

## Figures and Tables

**Table 1:** Concentrations of BMAA and DAB in mollusk animals analyzed in this study (μg·g^−1^ wet weight).

Sampling Location	Sampling Date	Mollusk Species	Toxin Concentration	
			BMAA	DAB
Beihai City	19 March 2016	*Atrina pectinata*	nd	nd
		*Crassostrea* sp.	nd	0.53
		*Perna viridis*	nd	0.87
		*Antigona lamellaris*	nd	0.26
		*Meretrix lusoria*	nd	nd
		*Periglypta petechialis*	nd	0.16
Zhoushan City	22 March 2016	*Crassostrea* sp.	nd	0.23
		*Mytilus coruscus*	0.45	1.61
		*Mytilus galloprovincialis*	nd	0.71
Rongcheng City	23 March 2016	*Crassostrea* sp.	nd	0.23
		*Chlamys farreri*	nd	0.81
		*Mytilus galloprovincialis*	nd	0.50
		*Solen strictus*	0.66	nd
		*Mactra chinensis*	nd	0.47
		*Ruditapes philippinarum*	nd	0.30
		*Neverita didyma*	2.15	0.32
Ningde City	24 March 2016	*Atrina pectinata*	nd	nd
		*Crassostrea* sp.	nd	0.61
		*Mytilus galloprovincialis*	nd	0.41
		*Sinonovacula constricta*	nd	0.17
		*Tegillarca granosa*	nd	nd
		*Periglypta petechialis*	nd	nd
		*Haliotis discus hannai*	nd	0.25
		*Turritella bacillum*	nd	nd
		*Natica maculosa*	nd	0.15
		*Batillaria zonalis*	nd	nd
		*Ruditapes philippinarum*	nd	0.74
Wenzhou City	25 March 2016	*Crassostrea* sp.	nd	0.70
		*Mytilus coruscus*	nd	0.63
		*Sinonovacula constricta*	nd	0.39
		*Tegillarca granosa*	nd	0.18
		*Turritella bacillum*	nd	nd
		*Moerella iridescens*	nd	0.051
Dalian City	25 March 2016	*Atrina pectinata*	nd	0.35
		*Crassostrea* sp.	nd	0.22
		*Chlamys farreri*	nd	0.67
		*Mytilus galloprovincialis*	nd	0.29
		*Scapharca subcrenata*	nd	0.55
		*Mactra chinensis*	nd	0.57
		*Ruditapes philippinarum*	nd	0.57
		*Volutharpa ampullacea*	nd	2.65
		*Neptunea cumingii*	nd	0.54
		*Neverita didyma*	3.97	0.21
Lianyungang City	26 March 2016	*Crassostrea* sp.	nd	0.83
		*Chlamys farreri*	nd	1.12
		*Mytilus galloprovincialis*	nd	0.58
		*Sinonovacula constricta*	nd	2.01
		*Arca inflata*	nd	0.24
		*Mercenaria mercenaria*	nd	0.17
		*Ruditapes philippinarum*	nd	0.71
		*Turritella bacillum*	nd	nd
		*Rapana venosa*	nd	nd
		*Neverita didyma*	0.99	0.40
Qinhuangdao City	26 March 2016	*Crassostrea* sp.	nd	0.45
		*Argopecten irradians*	nd	nd
		*Mytilus galloprovincialis*	nd	0.74
		*Rapana venosa*	nd	0.29
		*Arca inflata*	nd	1.02
Shenzhen City	28 March 2016	*Crassostrea* sp.	nd	nd
		*Mimachlamys nobilis*	nd	nd
		*Perna viridis*	nd	0.31
		*Gafrarium tumidum*	nd	1.00
Qingdao City	31 March 2016	*Crassostrea* sp.	nd	0.31
		*Chlamys farreri*	nd	1.07
		*Mytilus galloprovincialis*	nd	0.60
		*Haliotis discus hannai*	nd	nd
		*Rapana venosa*	nd	0.16
		*Ruditapes philippinarum*	nd	0.70
Laizhou City	26 April 2016	*Neverita didyma*	0.86	0.30
Qingdao City	22 May 2016	*Neverita didyma*	1.43	0.39

Note: nd—toxin concentration was lower than the limit of detection.

**Table 2:** Intensity percent of selective reaction monitoring (SRM) transitions (%) and retention times (min) of neurotoxins in different solutions.

		Standard Solution			Spiked Sample			Sample Extract	
		BMAA	DAB	AEG	BMAA	DAB	AEG	BMAA	DAB
Transitions	119 → 44	41.6 ± 0.7	12.5 ± 0.3	15.1 ± 0.3	40.7	11.5	15.6	40.4 ± 0.5	12.1 ± 0.6
	119 → 56	8.4 ± 0.2	21.9 ± 0.2	32.4 ± 0.2	9.3	22.0	32.0	10.1 ± 0.6	21.8 ± 1.6
	119 → 88	10.3 ± 0.2	0	0	10.1	0.0	0.0	10.1 ± 0.6	0
	119 → 101	0	43.6 ± 0.2	2.4 ± 0.2	0.0	44.2	2.6	0	44.7 ± 1.0
	119 → 102	39.7 ± 0.3	22.1 ± 0.3	50.1 ± 0.3	39.9	22.3	49.8	39.4 ± 0.9	21.4 ± 1.2
Retention times		15.33	16.27	16.97	14.36	15.01	15.67	14.32	14.99

## Supplementary Materials

The following are available online at www.mdpi.com/1660-3397/14/11/202/s1, Table S1: Summary of the analytical results of BMAA for aquatic organisms in previous studies, Table S2: Contents of amino acids detected in muscle and gland tissues of *Neverita didyma* (g/100 g wet weight).
