# Supplementary material for: New Typical Vector of Neurotoxin β-N-Methylamino-l-Alanine (BMAA) in the Marine Benthic Ecosystem
Source: Mar Drugs. 2016 Nov 4;14(11):202. doi: 10.3390/md14110202 (PMC5128745; doi:10.3390/md14110202)
Supplement: Supplementary file 1 [file marinedrugs-14-00202-s001.docx]

Supplementary Materials: New Typical Vector of Neurotoxin
β-*N*-methylamino-l-alanine (BMAA) in Marine Benthic Ecosystem

Aifeng Li, Jialiang Song, Yang Hu, Longji Deng, Ling Ding and Meihui Li

**Table S1.** Summary of the analytical results of BMAA for aquatic organisms in previous studies.

| **Aquatic Organism** | **Sampling Location** | **Content of BMAA** | **AQC** | **Detection System** | **Reference** |
| --- | --- | --- | --- | --- | --- |
| *Nostoc* sp. (CMMED-01) | Kaneohe Bay, on the island of Oahu in Hawaii | 10 µg/g or 25 µg/g | Yes | Amino Acid Analyzer,  HPLC-FD, UPLC-UV,  LC/MS, LC-MS/MS | [18] |
| 18 cyanobacterial strains | Portuguese estuaries,  Minho, Douro and Vouga. | 0.04~63 µg/g | Yes | HPLC-FD | [19] |
| 12 samples of cyanobacterial  blooms, scums and mats | 11 freshwater lakes and 1 lagon | 8~287 µg/g | Yes | HPLC-FD, LC-MS/MS | [20] |
| 21 spherical colonies of  *Nostoc commune* | Markets in Cusco, Peru | 6.18~18.9 µg/g | Yes | HPLC-FD, UPLC-UV,  UPLC/MS, LC-MS/MS | [21] |
| Dinoflagellate  (*Heterocapsa triquetra*) | Conservation in Ulla Rasmussen,  Stockholm University | Positive (not quantify) | Yes | LC-MS/MS | [16] |
| 5 species of marine diatom | Swedish west coast | 1.07~3.28 µg/g | Yes | UPLC-MS/MS | [17] |
| Shellfish, shrimp, and fish | Florida Bay, USA | nd~7000 µg/g | Yes | LC-MS/MS | [22] |
| Cyanobacteria,  shellfish, and fish | Southern archipelago of  Stockholm, Sweden | Cyanobacteria: 0.001~0.015 µg/g,  zooplankton: 0.024~0.087 µg/g,  fish: nd~1.29 µg/g, shellfish: 0.006~0.201 µg/g | Yes | LC-MS/MS | [23] |
| Mussel, oyster | Thau lagoon, France | 0.6~6.0 µg/g | Yes | HPLC-FD, UPLC-UV, UPLC/MS | [24] |
| Oyster, blue crab | Louisiana, Mississippi, and Florida | 5~47 µg/g | Yes | HPLC-FD, LC-MS/MS | [25] |
| Products of shark  fin and cartilage | South Florida, USA | Fin: 144~1836 ng/mg, cartilage: 86~265 µg/g | Yes | HPLC-FD, LC-MS/MS | [26,27] |
| Shellfish, dinoflagellate  (*Gymnodinium catenatum*) | Ria de Aveiro and Ria Formosa | Shellfish: nd~0.434 µg/g | Yes | UPLC-MS/MS | [28] |
| Cyanobacteria, shellfish, fish | Gonghu Bay of Lake Taihu, China | Cyanobacteria: 2.03~7.14 µg/g, shellfish:  0.12~8.76 µg/g, fish: 0.07~35.9 µg/g | Yes | LC-MS/MS | [29] |
| Plankton, fish | Lake Finjasjön in southern Sweden | Plankton: nd~6 ng/g, fish: 0.08~22.02 ng/g | Yes | UPLC-MS/MS | [30] |
| Cyanobacteria | Baltic Sea | nd | No | HILIC-MS/MS | [31] |
| Cyanobacteria | Water environments in China,  Germany, Brazil, South Africa, etc. | nd | No | HILIC-MS/MS | [32] |
| Cyanobacteria | Freshwater lakes of China | nd | No | HILIC-MS/MS | [33,34] |
| Cyanobacteria,  mussel, oyster | Thau lagoon, France | Cyanobacteria: nd, shellfish: 0.6~14.4 µg/g | No | HILIC-MS/MS | [35] |
| Mussel | Mussel used for reference materials | 0.95~1.2 µg/g | No | HILIC-DMS-MS/MS | [36] |
| Diatom, mussel, oyster | Coast of France | Diatom: nd~0.75 µg/g, shellfish: 0.2~6.7 µg/g | No | HILIC-MS/MS | [37,38] |

Note: AQC—derivatization using AQC; nd—content was lower than the limit of detection; HILIC-DMS-MS/MS: differential mobility spectrometry (DMS) as an ion filter to improve selectivity of LC-MS/MS.

**Table S2.** Contents of amino acids detected in muscle and gland tissues of *Neverita didyma* (g/100 g wet weight).

| **Amino Acid** | **DL-Muscle** | **DL-Gland** | **RC-Muscle** | **RC-Gland** | **LZ-Muscle** | **LZ-Gland** | **Muscle Average** | **Gland Average** |
| --- | --- | --- | --- | --- | --- | --- | --- | --- |
|  | **3.97 µg BMAA/g** | | **2.15 µg BMAA/g** | | **0.86 µg BMAA/g** | |  |  |
| Ala | 1.57 | 1.98 | 1.50 | 1.81 | 1.61 | 1.34 | 1.56 ± 0.06 | 1.71 ± 0.33 |
| Arg | 1.49 | 1.32 | 1.56 | 1.35 | 1.57 | 1.04 | 1.54 ± 0.04 | 1.24 ± 0.17 |
| Gly | 1.45 | 1.07 | 1.47 | 1.20 | 1.36 | 0.94 | 1.43 ± 0.06 | 1.07 ± 0.13 |
| Asp | 1.40 | 1.96 | 1.45 | 2.10 | 1.54 | 1.86 | 1.46 ± 0.07 | 1.97 ± 0.12 |
| Glu | 1.21 | 1.41 | 1.25 | 1.46 | 1.30 | 1.12 | 1.25 ± 0.05 | 1.33 ± 0.18 |
| Leu | 1.15 | 1.68 | 1.20 | 1.68 | 1.28 | 1.45 | 1.21 ± 0.07 | 1.60 ± 0.13 |
| Lys | 1.08 | 1.58 | 1.11 | 1.56 | 1.19 | 1.17 | 1.13 ± 0.06 | 1.44 ± 0.23 |
| Ser | 0.93 | 1.27 | 1.00 | 1.33 | 1.04 | 1.24 | 0.99 ± 0.06 | 1.28 ± 0.05 |
| Val | 0.70 | 1.15 | 0.70 | 1.11 | 0.77 | 1.09 | 0.72 ± 0.04 | 1.12 ± 0.03 |
| Pro | 0.64 | 0.69 | 0.65 | 0.79 | 0.65 | 0.84 | 0.65 ± 0.01 | 0.77 ± 0.08 |
| Thr | 0.58 | 0.92 | 0.61 | 0.97 | 0.65 | 0.91 | 0.61 ± 0.04 | 0.93 ± 0.03 |
| Phe | 0.57 | 1.02 | 0.57 | 0.98 | 0.63 | 0.90 | 0.59 ± 0.03 | 0.97 ± 0.06 |
| Ile | 0.57 | 0.94 | 0.58 | 0.90 | 0.62 | 0.74 | 0.59 ± 0.03 | 0.86 ± 0.11 |
| Tyr | 0.49 | 0.78 | 0.47 | 0.77 | 0.52 | 0.80 | 0.49 ± 0.03 | 0.78 ± 0.02 |
| Met | 0.38 | 0.55 | 0.43 | 0.50 | 0.46 | 0.49 | 0.42 ± 0.04 | 0.51 ± 0.03 |
| His | 0.27 | 0.47 | 0.27 | 0.45 | 0.30 | 0.39 | 0.28 ± 0.02 | 0.44 ± 0.04 |

Note: “DL” refers to the sample collected from Dalian city; “RC” refers to the sample collected from Rongcheng city; and “LZ” refers to the sample collected from Laizhou city.
